# The Effect of Metal Shielding Layer on Electrostatic Attraction Issue in Glass–Silicon Anodic Bonding

**DOI:** 10.3390/mi16010031

**Published:** 2024-12-28

**Authors:** Wenqi Yang, Yong Ruan, Zhiqiang Song

**Affiliations:** 1 Instrument Science and Opto-Electronics Engineering, Beijing Information Science and Technology University, Beijing 100192, China; 15904233980@163.com; 2 Department of Precision Instruments, Tsinghua University, Beijing 100084, China; 3 Zhejiang Xinsheng Semiconductor Technology, Zhuji 311899, China; songzhiqiang@chipright.com.cn

**Keywords:** microelectromechanical systems (MEMS), wafer-level packaging, anodic bonding, electrostatic attraction, design and optimization

## Abstract

Silicon–glass anode bonding is the key technology in the process of wafer-level packaging for MEMS sensors. During the anodic bonding process, the device may experience adhesion failure due to the influence of electric field forces. A common solution is to add a metal shielding layer between the glass substrate and the device. In order to solve the problem of device failure caused by the electrostatic attraction phenomenon, this paper designed a double-ended solidly supported cantilever beam parallel plate capacitor structure, focusing on the study of the critical size of the window opening in the metal layer for the electric field shielding effect. The metal shield consists of 400 Å of Cr and 3400 Å of Au. Based on theoretical calculations, simulation analysis, and experimental testing, it was determined that the critical size for an individual opening in the metal layer is 180 μm × 180 μm, with the movable part positioned 5 μm from the bottom, which does not lead to failure caused by stiction due to electrostatic pull-in of the detection structure. It was proven that the metal shielding layer is effective in avoiding suction problems in secondary anode bonding.

## 1. Introduction

Packaging, which is crucial for ensuring the reliability of MEMS devices, is an important step in the fabrication process of Micro-Electro-Mechanical Systems (MEMS). Research indicates that MEMS devices, including micro-resonators and micro-accelerometers [1,2,3], have small movable structures that are significantly affected by environmental factors such as air damping and humidity [4]. Proper packaging can effectively reduce the impact of external factors. Typically, MEMS devices operate under high-temperature and high-pressure conditions, and there are movable structures inside that facilitate the transmission of electrical, optical, and mechanical quantities. Therefore, certain mechanical protection for the bare die is necessary to shield the devices from external environmental interference. Wafer-level packaging plays a vital role in ensuring the structural integrity and performance stability of MEMS devices.

One of the key factors affecting the quality of wafer-level packaging is the bonding condition between the cap and the substrate. The wafer-level packaging discussed in this paper achieves the bonding of the substrate and cap through silicon–glass anodic bonding. The anodic bonding method was first proposed by Wallis in 1969 [5], enabling a good connection between silicon and glass. It has been widely used in hermetic packaging and is a key technology in MEMS fabrication.

Anodic bonding technology primarily achieves sealing effects by forming chemical bonds through an electric field. In 2001, Veenstra [6] successfully realized selective anodic bonding by adding a metal anti-bonding layer of less than 1 nm on glass wafers, resulting in a micro-pump chamber with a diameter of 11 mm and no dead volume. In 2010, Hou [7] employed a stepwise pressure increase method to reduce the impact of electrostatic forces on the bonding structure while ensuring bonding strength, thereby improving residual stress during the bonding process and avoiding adhesion failure during bonding. In 2018, Wei [8] established and validated a pull-in effect model for analyzing and calculating the pull-in voltage during the anodic bonding process, verifying the effectiveness of the pull-in effect model through finite element analysis and experimental validation. In 2020, Hao [9] derived the electrostatic pull-in voltage formula for the gyroscope beam and glass and established a model relating the pull-in voltage to the gap between the silicon structure and glass. However, most of these studies focus on pull-in failure phenomena occurring during the first anodic bonding, with little research addressing the failure of the movable structures inside the cavity caused by the electrostatic forces generated during the cap–substrate connection voltage in the second anodic bonding.

**Figure 1:**
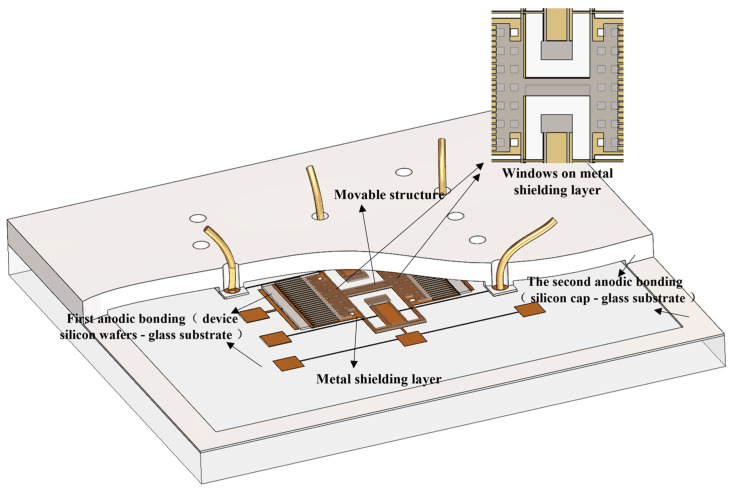
The overall structure of glass–silicon wafer-level packaging.

The wafer-level packaging technology studied in this paper utilizes two types of anodic bonding. The overall structure of the wafer-level packaging studied in this paper is shown in Figure 1. The primary focus of this study is on methods to prevent pull-in failure of internal movable structures during the second anodic bonding process between the cap and the substrate. Moreover, in order to reduce the complexity of the subsequent de-capping test and facilitate the direct observation of the device through the side of the substrate, this study investigates the design of windows in the metal shielding layer, focusing on the design of window dimensions. To address the issue of device failure at the window, the effect of electrostatic forces on the movable structure connected by cantilever beams during the second anodic bonding is modeled as the electrostatic attraction between parallel plate capacitors. Theoretical calculations and simulation analysis methods were developed, which can serve as a reference for standardized design rules in future processes.

## 2. Electrostatic Pull-In Phenomenon

### 2.1. The Principle of Anodic Bonding

**Figure 2:**
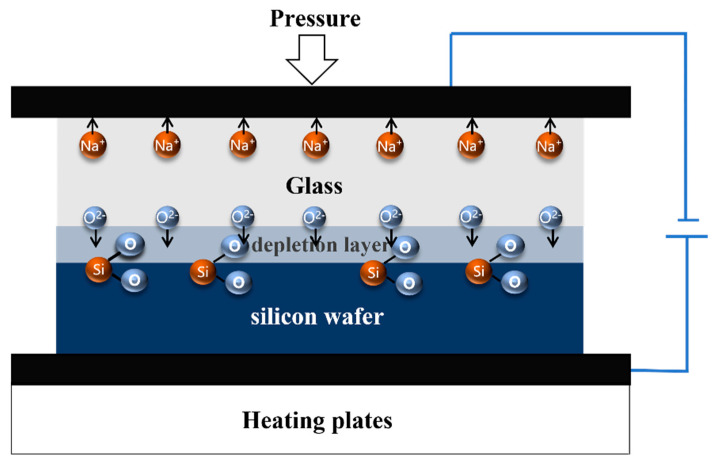
Silicon–glass anodic bonding.

A schematic diagram of the anode bonding process is shown in Figure 2, where the silicon wafer and the glass are overlapped and sandwiched between two electrodes, the glass is connected to the cathode, and the silicon wafer placed on a heating plate is connected to the anode. The hot plate provides the required temperature for the bond. The mobility of sodium ions in the glass increases with the increase in temperature, and when the temperature rises to the bonding temperature, a depletion layer will be formed near the silicon–glass interface. At the same time, the oxygen ions in the glass move to the silicon, and a positive charge is also induced on the surface of the silicon wafer. Under the interaction of positive and negative charges, a strong adsorption force is formed, which causes the silicon wafer and the glass to be in close contact to produce a small deformation, and the anodic oxidation reaction occurs at the silicon–glass interface to form a strong chemical bond O-Si-O [10]. The silicon wafer used in our experiment has a low resistivity of 0.002~0.005 Ω·cm, and the glass used is *BF33*.

### 2.2. Electrostatic Pull-In Failure

In wafer-level packaging, during the first anodic bonding, the silicon wafer is bonded to the glass substrate, followed by thinning and etching processes to create the device structure. The silicon wafer has high rigidity, so it is not affected by electrostatic forces and does not adhere to the substrate. However, during the second anodic bonding process, an 800 V voltage is applied between the cap and the substrate. This causes movable structures inside the device, such as micro-cantilevers, to experience electrostatic pull-in failure to the substrate. In the high-temperature and high-pressure environment (350 °C, 700 N), these structures adhere to the substrate, resulting in the failure of the movable parts.

To address this issue, a metal shielding layer was designed to reduce the electric field intensity between the device and the substrate, thereby suppressing the electrostatic pull-in phenomenon. The metal shielding layer is required to reduce the electric field intensity between the device and the substrate during the secondary anodic bonding process while ensuring that the shielding layer does not affect the normal operation of the device, particularly the electrical signal transmission and structural stability. Additionally, it must meet the manufacturing process requirements to avoid increasing the process complexity due to the shielding layer’s design. Considering that the bottom structure of some devices may have specific requirements for electrical connection or optical performance (e.g., the substrate must be perforated or transparent), the design of the metal shielding layer needs to balance the practical requirements of both full coverage and windowed configurations.

**Figure 3:**
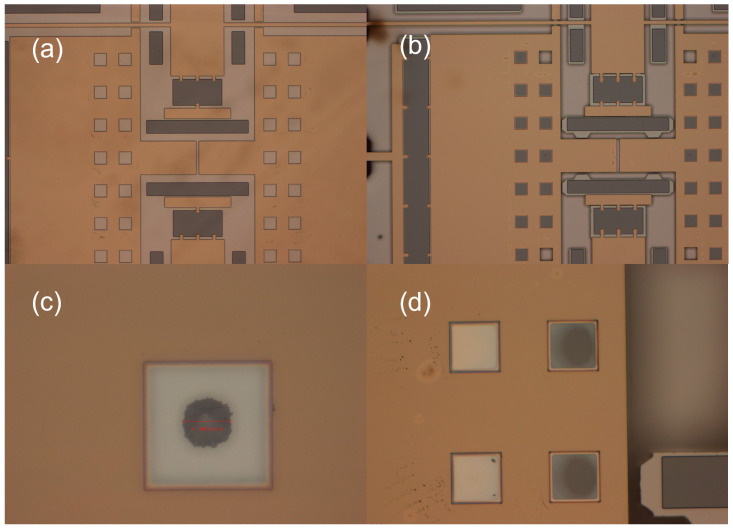
(**a**) Glass sheet before secondary bonding; (**b**) glass sheet after secondary bonding; (**c**) morphology at a single window; (**d**) morphology at windows.

Figure 3 shows images illustrating the failure occurring at the window, observed under a microscope at a magnification of 31.5×. The images in (c) and (d) are cropped from the overall image to highlight specific areas. During the process implementation, as shown in Figure 3a, no failure was observed at the window after the first anodic bonding when observed through the substrate. After the second anodic bonding, the structure of the device was observed in Figure 3b, and under this structure, the first anodic bonding did not cause failure in the movable structures. The failure mainly occurred during the second anodic bonding process. As shown in Figure 3c,d, by observing the failure at the window under a microscope, it was evident that the failure was due to the device adhering to the substrate at the window. Based on the color change, it was determined that the silicon device had bonded with the substrate at the adhered area. After de-capping the chip following the second bonding, the probe station was used to manipulate the movable structure with a probe. It was found that the structure could not be moved by the probe, indicating failure in the movable structure, rendering it non-functional.

### 2.3. Pull-In Voltage Analysis

**Figure 4:**
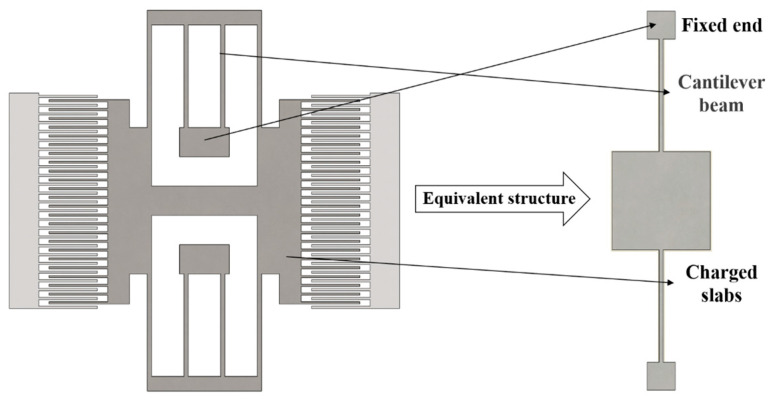
A simple detection structure equivalent to the resonator structure.

The movable part structure of the device is fixedly connected by four folding beams, which can be regarded as the structure of an intermediate mass connected by a cantilever beam. The complex structure of the device is simplified, and the simple detection structure is designed. The equivalent structure is shown in Figure 4. The design is simple and the structural dimensions of the double-end fixed cantilever beam are shown in Table 1.

In MEMS, beams are elastic elements, and the stress state of the beam can be modeled as a mechanical spring. The relationship between displacement and external force follows the linear relationship expressed by Hooke’s law. The mechanical elastic deformation constant is the ratio of the external force to the displacement it causes [11]:

(1) $$k_{m}=\frac{F}{x}=\frac{12EI}{l^{3}}=\frac{Ewt^{3}}{4l^{3}}$$

Among them, $F_{m}$ is the force acting on the beam, x is the deflection of the cantilever beam, I is the moment of inertia of the cross-section of the cantilever beam $I=\frac{wt^{3}}{12}$, w is the width of the beam, t is the thickness of the beam, and l is the length of the beam.

The equivalent mechanically elastic deformation constant of the slab supported by two fixed-guide beams is:

(2) $$k_{m\_total}=\frac{2Ewt^{3}}{4l^{3}}=\frac{Ewt^{3}}{2l^{3}}$$

The elastic modulus of silicon is E=170GPa. After calculation, the equivalent mechanical elastic deformation constant of this detection structure is:

(3) $$k_{m\_total}=\frac{Ewt^{3}}{2l^{3}}=\frac{170\times 10^{9}\times 10\times 10^{-6}\times {\left(25\times 10^{-6}\right)}^{3}}{2\times {\left(800\times 10^{-6}\right)}^{3}}\approx 25.94$$

In static equilibrium, the mechanical restoring force is equal to the magnitude of the electrostatic force and in opposite directions, and the effective spring constant of the structure is the mechanical spring constant minus the electric spring constant. Assuming that the vertical displacement is x at equilibrium, the distance between the electrodes becomes $d+x\left(d+x<d\right)$. At electrostatic equilibrium, the electrostatic force is:

(4) $$F_{e}=\frac{1}{2}\frac{\varepsilon A}{{\left(d+x\right)}^{2}}U^{2}=\frac{1}{2}\frac{C(x)U^{2}}{\left(d+x\right)}$$

Among them, ε is the vacuum permittivity, A is the effective electrode area of the plates, U is the voltage, and d is the distance between the electrodes.

The spatial gradient of the electrostatic force is defined as the electric spring constant:

(5) $$k_{e}=\left|\frac{\partial F_{e}}{\partial d}\right|=\left|-\frac{CU^{2}}{d^{2}}\right|=\frac{CU^{2}}{d^{2}}$$

The mechanical restoring force is:

(6) $$F_{m}=-k_{m}x$$

Based on Equations (4) and (5), we obtain:

(7) $$U^{2}=\frac{2k_{m}x{\left(x+d\right)}^{2}}{\varepsilon A}=\frac{2k_{m}x(x+d)}{C}$$

*x* satisfies the unique solution:

(8) $$x=-\frac{d}{3}$$

The pull-in voltage is obtained as:

(9) $$U_{p}=\frac{2d}{3}\sqrt{\frac{k_{m}}{1.5C_{0}}}=\frac{2d}{3}\sqrt{\frac{k_{m}d}{1.5\varepsilon A}}$$

where $C_{0}$ is the static capacitance of the device:

(10) $$C_{0}=\frac{\varepsilon A}{d}$$

When a metal shielding layer is added along with the window, the area of the window can be equivalently treated as the area exposed in the parallel plate configuration, allowing the pull-in voltage to be calculated for different window sizes.

## 3. Simulation Analysis

To ensure that the device functions normally after packaging, electrical connections must be established through the metal pads on the substrate. During the second bonding process, applying voltage to the cap creates an electrical connection, increasing the potential on the device. This results in a downward electrostatic force, causing it to pull in and adhere to the substrate, leading to failure. However, adding a metal shielding layer on the substrate reduces the potential difference between the device and the substrate, preventing the pull-in issue. After creating a window, there is no longer a metal shielding layer between the device corresponding to the window position and glass substrate, resulting in a potential difference between the device and the substrate. As the window size increases, this potential difference also increases. Finite element analysis has yielded similar results.

**Figure 5:**
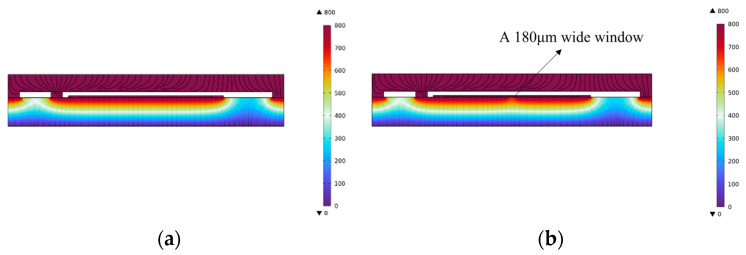
Simulation of electric potential (**a**) without a window and (**b**) with a 180 μm × 180 μm window.

**Figure 6:**
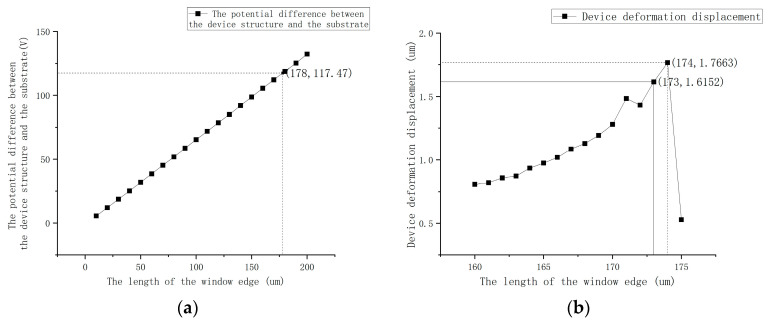
(**a**) The relationship between the window edge length and the potential difference (V) between the device and the substrate. (**b**) The relationship between the window edge length and the device deformation size.

Figure 5 shows the simulation comparison of the electric field variation with and without windows in the metal layer. The material of the metal layer is set to gold (Au), with a thickness of 400 Å. The voltage on the upper surface of the structure is set to 800 V and on the bottom surface is set to 0 V. Figure 5b shows the voltage of the overall structure when a 180 μm-width notch is added to the metal layer using COMSOL Multiphysics^®^ version 6.1. Through simulation analysis of the electric potential field of the overall structure, the influence of the window edge length on the potential difference between the device and the substrate, as well as the device deformation, was studied. Figure 6a shows the relationship between the window edge length and the potential difference between the device and the substrate, while Figure 6b presents the corresponding curve of the window edge length versus the device deformation. The simulation results show that the size of window in the metal layer has an effect on the potential difference between the device and the substrate. As the window edge length increases, the potential difference between the device and the substrate increases linearly. When the window edge length reaches a certain value, the electrostatic force acting on the device exceeds its mechanical restoring force, leading to electrostatic pull-in. Two-dimensional simulations have limitations. Therefore, to better observe trends, three-dimensional simulations were employed. In the three-dimensional modeling under the potential field, the voltage conditions for different window edge lengths are simulated, and the potential at the center of the substrate is measured. This results in a curve illustrating the relationship between the window edge length and the potential.

Under the influence of the electric current field, the potential of the monolithic structure was conducted. The calculations yielded a graphical representation of the relationship between the window edge length and the potential difference between the device and the substrate. It can be observed that the potential difference increases linearly with the increase in window edge length. The effect of window edge length on the degree of device engagement can be calculated by the value obtained by the simulation. The window area can be equivalently treated as the area of the device affected by the electric field. By substituting this into Equation (8), the pull-in voltage corresponding to the window area is obtained. Comparing the pull-in voltage with the potential difference between the device and the substrate at the window position, it is determined that the device will pull in when the window edge length exceeds 178 μm. The calculation formula is:

(11) $$U_{p}=\frac{2d}{3}\sqrt{\frac{k_{m}d}{1.5\varepsilon A}}=\frac{2\times 5\times 10^{-6}}{3}\sqrt{\frac{25.94\times 5\times 10^{-6}}{1.5\times 8.854\times 10^{-12}\times {\left(178\times 10^{-6}\right)}^{2}}}=58.52V$$

**Figure 7:**
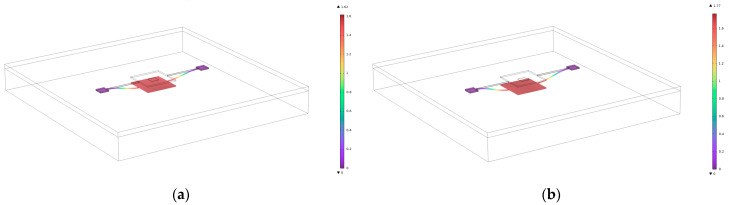
(**a**) Displacement of the electrostatic force of the device with a window size of 173 μm; (**b**) displacement of the device with an electrostatic force at a window size of 174 μm.

Under the electrostatic field, the same potential difference is set, and the displacement of the device under the influence of the electric field is calculated for each window edge length and voltage difference. This results in a graphical representation of the relationship between window edge length and device deformation displacement. According to Equation (8), when the displacement exceeds 1.667 μm, the device will pull in. As indicated in the annotation of Figure 6b, the critical window size leading to pull-in, determined using the two-point method, lies between 173 μm and 174 μm. Specifically, the key results obtained from the simulation are as follows:

As shown in Figure 7a, when the window edge length is 173 μm, the maximum displacement of the device under the electrostatic force is approximately 1.62 μm, which does not reach the pull-in condition.

As shown in Figure 7b, when the window edge length increases to 174 μm, the maximum displacement of the device reaches 1.77 μm, indicating that the device has reached the critical state of pull-in.

When the window edge length further increases to 175 μm, the device undergoes significant displacement in the simulation, confirming the existence of a critical window size.

In conclusion, to effectively suppress the electrostatic pull-in failure, the window edge length should be controlled below 173 μm to ensure stable operation of the device. This also provides clear guidance for the design of shielding layer windows.

The error between the simulation results and theoretical calculations is approximately 2.2%, which may be attributed to the idealized boundary conditions and the precision of mesh division in the simulation. Furthermore, the theoretical calculation assumes that the elastic restoring force of the device follows an ideal linear relationship, whereas the actual mechanical characteristics of the device may exhibit nonlinear effects, particularly near the critical state of pull-in, where the material may show more complex elastic deformation behavior. As a result, there is a certain discrepancy between theoretical calculations and finite element simulations. However, overall, the simulation results are in good agreement with the theoretical calculations, verifying the influence of window edge length on potential distribution and electrostatic pull-in.

## 4. Experiments and Discussion

### 4.1. Fabrication of Metal Layers

To address the issue of device structural failure caused by electrostatic pull-in failure, an Au/Cr metal layer was added to the glass substrate as a solution. The main processes were as follows: (1) Photolithography was performed on the cleaned glass wafer; (2) a 3000 Å-deep trench was etched using BOE etching technology, and the photoresist was removed from the glass wafer; and (3) a 400 Å-thick layer of Cr and a 3400 Å-thick layer of Au were sputtered, and metal patterning was achieved using lift-off technology after photolithography.

### 4.2. Detection Structure Design

**Figure 8:**
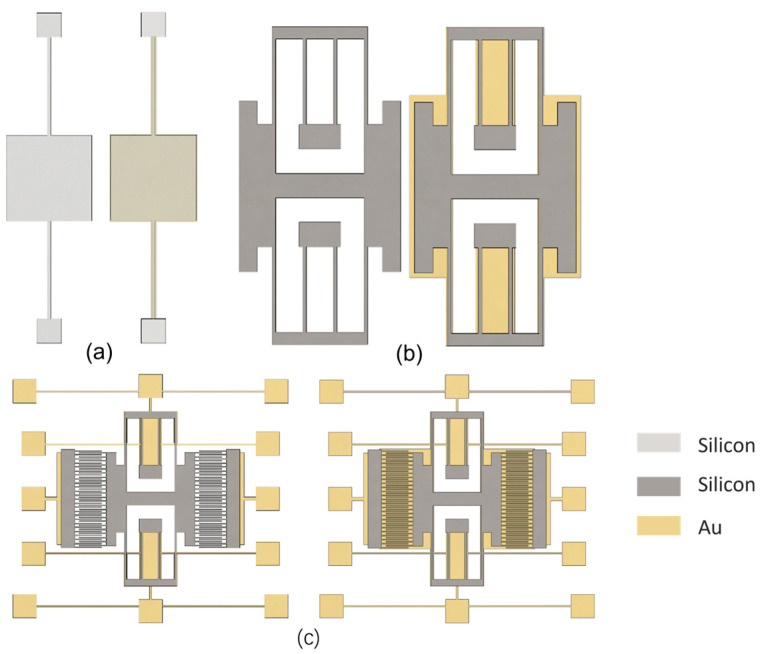
Structure without a metal shield and with a metal shield (**a**); simple detection structure (**b**); movable part without comb fingers (**c**).

The device was designed with a metal barrier layer added at the bottom and without the metal barrier layer. The metal barrier layer completely covered the device structure on the substrate to verify whether the addition of the metal shielding layer could effectively prevent the pull-in issue during the second anodic bonding process. The detection structure is shown in Figure 8.

### 4.3. Verify the Role of Full Coverage of Metal Shielding

**Figure 9:**
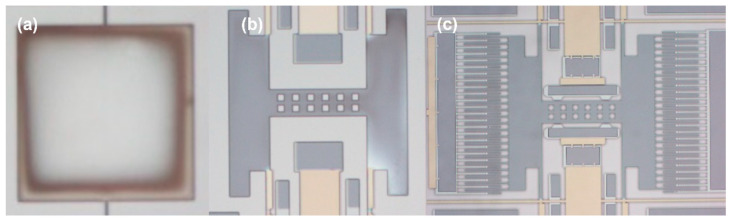
Morphologies of (**a**) the simple structure, (**b**) the comb-free movable structure, and (**c**) the resonator structure after secondary bonding without a metal shield.

**Figure 10:**
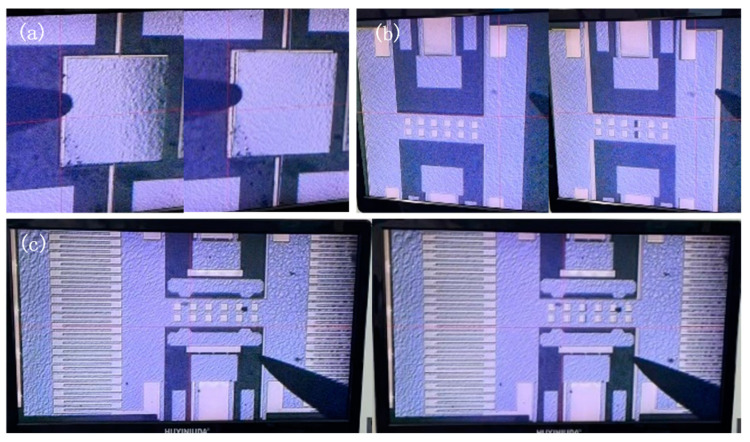
Structures with the addition of a metal shield after secondary bonding: before being pushed and after being pushed by a probe for (**a**) the simple structure, (**b**) the resonator structure without combs, and (**c**) the resonator structure with combs.

Since the device could not be seen through the metal layer due to observation through the glass substrate, we opened the packaged chip and used a probe to push the plate. By observing under a microscope whether the plate could be moved, we determined whether it had adhered due to pull-in. The morphology of three structures without a metal shielding layer after the second anodic bonding process is shown in Figure 9 under a 63× magnification using a microscope. Experimental results show that failure occurred in simple structures, structures without comb fingers, and resonator structures when no metal shielding layer was added. By using a probe to gently push the movable part of the structure, it was observed whether it could move, thereby determining whether the device experienced pull-in failure, as shown in Figure 10. After adding the metal shielding layer, the structures could be moved by the probe. The experimental results indicate that the addition of the metal shielding layer effectively prevented device failure during the second bonding process.

### 4.4. The Influence of Window Size on Device Failure

Previous experiments have verified that placing a metal layer on the bottom of the device can effectively prevent the device from pulling in the substrate during the anodic bonding process. However, on devices where the bottom is not suitable for the bottom metal layer, there should be a gap in the metal layer added to the substrate. For such cases, the effect of the size of the window in the metal layer in the engagement situation was investigated.

**Figure 11:**
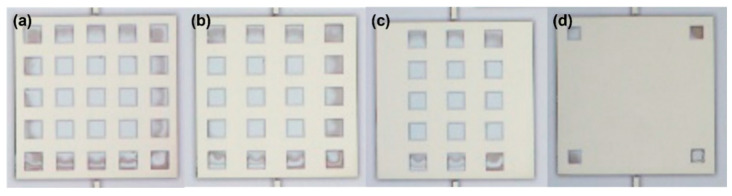
Observation of the different quantities and morphologies of metal-layer windows through the glass substrate side: (**a**) 25 windows, (**b**) 20 windows, (**c**) 15 windows, (**d**) 4 windows.

As shown in Figure 3, in the packaged comb resonator structure, when the window edge length of the metal layer was 80 μm, the movable structure of the device experienced pull-in. The glass exposed at the window position bonded with the movable structure of the resonator, causing the movable structure to adhere to the substrate, resulting in device failure. A doubly clamped beam structure equivalent to the original resonator’s movable structure was designed. For the metal layer with a window edge length of 80 μm, comparative experiments were conducted with different numbers of windows, specifically testing structures with 25, 20, 15, 6, and 4 windows. The fabrication process was the same as described in Section 4.1. Figure 11 shows the morphology observed from the glass side after the second anodic bonding.

**Figure 12:**
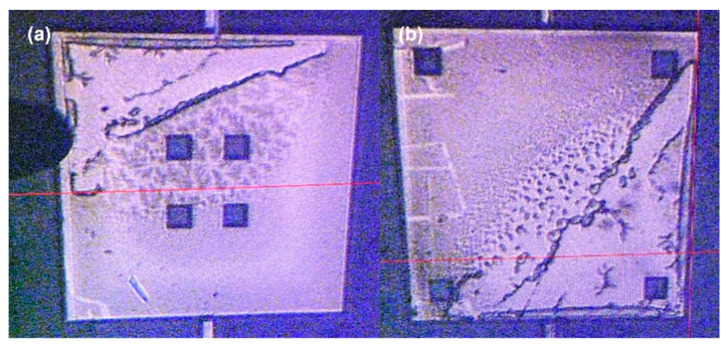
The black substance on the metal layer after secondary anodic bonding. (**a**) Structure with 4 windows in the center (**b**) Structure with 4 windows around the edges

After opening the packaged chip, it was observed that there was a suspected failure of black material in some areas between the device structure and the metal through the microscope, as shown in Figure 12, with the image magnified 10 times on the probe station. However, after the probe station toggled the device structure, the black area was not firmly bonded, and it was not directly bonded to the glass substrate like the detection structure at the previous window. When lightly scraping the glass surface with a probe, black substances were found on the surface. After consulting the literature [6], it was determined that these substances were alkaline compounds generated during the anodic bonding process. When lightly pushing some of these test structures laterally, a slight resistance was felt, but they could still be moved. This suggests that the structures were slightly adhered due to the substances precipitated during the anodic bonding process rather than being completely pulled in.

**Figure 13:**
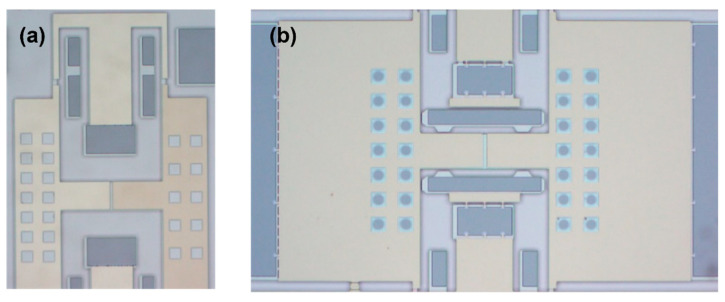
(**a**) Microscope view of the movable part of the comb-free structure from the substrate side; (**b**) microscope view of the resonator structure from the substrate side.

When the window edge length was 80 μm, the test structures with 25, 20, 15, 6, and 4 windows in the metal layer were laterally pushed and vertically pressed using a probe station, and no pull-in occurred. A comparison of the failure in the movable structure of the resonator without comb fingers and the overall resonator structure at the metal-layer window region is shown in Figure 13. The resonator structure with comb fingers experienced severe failure at the window after the second bonding process; the device layer at the window had bonded with the glass, and the entire movable structure could not be moved with the probe station. In contrast, the structures without comb fingers, even with more windows of the same size, did not experience pull-in. A comprehensive analysis of the pull-in behavior in the three types of test structures led to the conclusion that the extent of pull-in was related to the support strength of the cantilever beam. During the second bonding process, an electric field was generated between the cavity inside the cap and the substrate, exerting a downward force on the internal structure. However, due to differences in the support strength of the cantilever beam, the devices exhibited varying degrees of displacement. In regions with a metal layer, even if the device fully contacted the substrate, the metal prevented the bonding of silicon and glass. However, in the resonator structure, the cantilever beam supported a heavier structure, and the mechanical restoring force of the cantilever was insufficient to lift the entire structure back. As a result, anodic bonding occurred between the silicon and glass at the window region.

**Figure 14:**
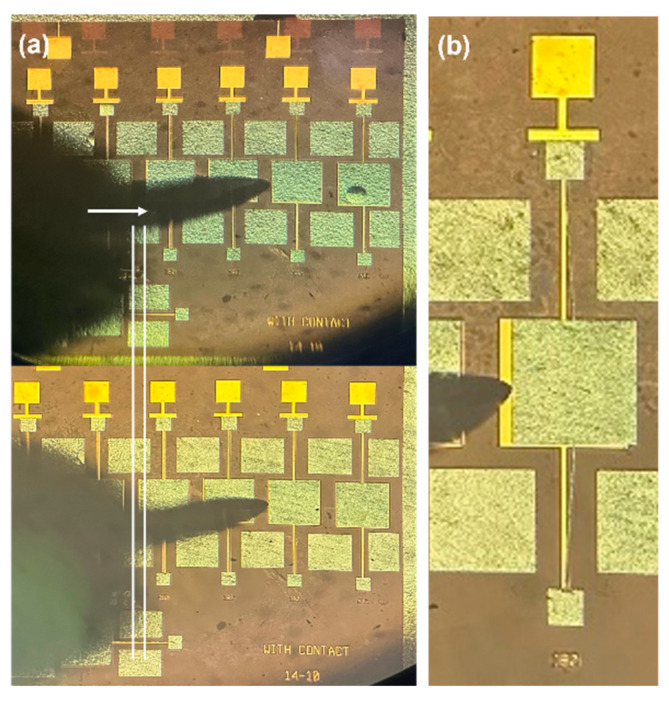
(**a**) The probe cannot be pushed when the window edge length is 190 μm; (**b**) the probe can be pushed when the window edge length is 180 μm. As shown by the arrow in the figure, the probe pushes forward, but the movable structure remains immobile.

Based on simulation and theoretical calculations, square windows with edge lengths ranging from 160 μm to 190 μm were designed. After the second anodic bonding, the internal test structures were evaluated. The movable parts of the test structures were manipulated using a probe on a probe station. The experimental results are shown in Figure 14. In Figure 14a, when the probe was used to push the device structure corresponding to a metal layer window with an edge length of 190 μm, the device could not be moved even after pushing a certain distance, indicating that the structure had failed. Figure 14b shows that the device structure corresponding to a window with an edge length of 180 μm could be easily moved by the probe. In the experiment, none of the test structures with window edge lengths smaller than 180 μm experienced pull-in to the substrate during or after the second bonding, which is consistent with the simulation and theoretical calculation results.

## 5. Conclusions

This paper investigates the addition of a metal shielding layer in silicon-on-glass wafer-level packaging to address the issue of movable part failure caused by the second anodic bonding process. The following conclusions were drawn:

1. It was verified that adding a metal layer beneath the movable structure can effectively prevent failure due to electrostatic forces during anodic bonding.
2. By designing simple test structures, the reasons why the metal layer prevents pull-in failure of the device were analyzed. First, the metal layer provides an electrical connection between the device and the bottom metal layer, eliminating the potential difference between the movable structure and the substrate, and thus preventing electrostatic forces from causing pull-in failure. Second, in the experiment, the distance between the bottom of the device and the substrate was only 5 μm. Since the cantilever beam can deform by 5 μm without breaking, when the device experiences excessive potential during the second bonding process, electrostatic forces cause the structure to contact the substrate. The Cr layer prevents silicon from reacting with the glass. After the second bonding, the cantilever beam’s mechanical restoring force eliminates electrostatic effects and protects the device from failure.
3. Due to uncertainties in the fabrication process and the varying shapes of device structures, it is difficult to quantitatively determine the limit size of the window. However, based on the calculation approach proposed in this paper, it is possible to estimate a suitable gap size. For various types of devices, particularly those for which it is not suitable to add a metal layer beneath the structure, the maximum window size can be preliminarily determined through calculation and simulation analysis when using a wafer-level packaging solution with second anodic bonding.
4. The overall error between the experiment, simulation, and theoretical calculation is within 2.25%, which is considered acceptable. The possible reasons for the error include the following:
5. Firstly, theoretical calculations assume that the mechanical response of the device material follows an ideal linear relationship. However, under high voltage conditions, the actual material may exhibit slight nonlinear characteristics, especially near the critical pull-in state. This nonlinear effect may contribute to the error.
6. Secondly, in the simulation process, the shielding layer is simplified as an ideal conductor, without considering the influence of surface roughness, oxide layers, and the uneven microscopic morphology of the device. This simplification may lead to discrepancies in the local electric field distribution compared to the actual case, thereby affecting the calculation of the critical window size.
7. Lastly, in the experiment, there may be certain deviations in the actual fabricated dimensions of the window edge length due to manufacturing precision limitations. These deviations may result in differences between the actual and designed window sizes.
8. Due to experimental limitations, the direct measurement of structural displacement remains challenging. Instead, this study relies on a combination of theoretical modeling, numerical simulations, and qualitative experimental validation to investigate the pull-in failure. The consistency between simulation and theoretical results provides strong evidence for the critical design parameters. Future work will focus on developing advanced measurement techniques to directly quantify the deformation for further refinement of the proposed design rules.

## Figures and Tables

**Table 1:** Detection of structure dimensions.

The Name of the Structure	Size (μm)
Length of the cantilever beam (l)	800
Width of the cantilever beam (w)	10
Thickness of the structure (t)	25
Distance between the bottom surface of the structure and the substrate (d)	5

## Data Availability

No new data were created.
